# Editorial: The Effects of Altered Gravity on Physiology

**DOI:** 10.3389/fphys.2019.01447

**Published:** 2019-11-27

**Authors:** Gilles Clement, Richard D. Boyle, Hanns-Christian Gunga

**Affiliations:** ^1^Lyon Neuroscience Research Center, Bron, France; ^2^National Aeronautics and Space Administration, Washington, DC, United States; ^3^Institute of Physiology, Charité Medical University of Berlin, Berlin, Germany

**Keywords:** adaptation, microgravity, hypergravity, spaceflight adaptation, extreme environment

In physiology, a graded dose-response curve relates the stimulus input to a specific measured output. Space studies in humans and animals have provided only a snapshot into understanding the role of gravity on physiological responses. Fully understanding this relationship, including adaptive mechanisms, will provide the information required to ensure normal physiological function in crew for long-duration space missions (Clément, 2017).

Various methods can be used for generating altered gravity, including orbital flight, parabolic flight, head down/up tilt, body loading/unloading, and centrifugation (Richter et al.). In this *Frontiers in Physiology* Research Topic, ten papers have used these various methods for studying the effects of altered gravity on sensorimotor, musculoskeletal, cardiopulmonary, and cerebrovascular responses in humans and rodents.

## Orbital Flight

Long stays in weightlessness take a toll on the human body, as the muscles atrophy, bones lose minerals, and a new set of stimuli imposes novel challenges on the vestibular and cardiovascular systems (Gunga et al., 2016). Stationary bike and treadmill were used on Sklylab and Mir stations; however, they were not completely successful in preventing astronauts' muscle and bone loss. A new exercise device, the Interim Resistive Exercise Device (iRED), was flown on the International Space Station to target weight-bearing structures, such as squats to load the spine, hips and legs (Schneider et al., 2003). McNamara et al. analyzed computed tomography scans of 16 crewmembers before and after long-duration spaceflight and found that crewmembers experienced less decrease in the back, abdominal, and paraspinal muscles after using iRED protocols in orbit.

During spaceflight, the vestibular otolith organs no longer adequately sense gravito-inertial accelerations. Animal studies have shown that otolith afferents are initially hypersensitive to tilt after return to Earth (Boyle et al., 2001). Perhaps as a result of this hypersensitivity, astronauts overestimate pitch and roll tilt for 1–2 days immediately after landing (Clément and Wood, 2013). In addition, their performance during manual motion-based simulation tasks is greatly impaired, which is a navigational concern (Moore et al., 2019). Clément et al. showed that adaptive changes in astronauts' vestibular processing during spaceflight impair their ability to manually control tilt following transitions between gravitational environments; however, simple aids, such as vibrotactile feedback, can be used to improve their performance.

These changes in vestibular processing after spaceflight are confirmed by brain imaging study showing functional connectivity modifications between the vestibular cortex, the vestibular nuclei, and the cerebellum in cosmonauts after long-duration spaceflight (Pechenkova et al.). Interestingly, the severity of space motion sickness symptoms experienced by these subjects was found to correlate with a post- to pre-flight difference in connectivity between the right and left areas of the vestibular cortex.

## Parabolic Flight

The parabolic trajectory of an aircraft exposes the passengers to repeated transitions from increased to reduced gravity (Karmali and Shelhamer, 2008). Ritzmann et al. compared postural adjustments in response to perturbations across gravity levels ranging from 0.25 to 1.75 g by varying the aircraft's pitch angle during pull-up. Subjects walked on a split treadmill and changes in their leg muscles activity and ankle and knee joint trajectories were recorded as the speed of the belts suddenly changed. Results showed a linear relationship between gravity level and EMG amplitudes and muscle onset latencies after perturbations.

## Head Down/Up Tilt

Body tilt of supine subjects allows for partial mechanical unloading along the subject's longitudinal axis in normal Earth's gravity and simulate cephalad fluid shifts (Pavy-Le Traon et al., 2007). Boschert et al. placed subjects at −12° (head down), corresponding to −0.2 g linear acceleration (Clément et al.), for 3 days and two nights to determine whether changes in cerebral hemodynamics also affected sleep. Results indicated that jugular vein venous congestion occurred faster and that the quality of sleep was poorer at the greater head tilt angle.

Diaz-Artiles et al. compared cardiopulmonary responses of subjects to various gravitational loads along the body longitudinal axis as they exercised on a cycle ergometer while supine with the head-down or head-up. Angles of tilt were −6° (simulating 0 g), 9.5° head up (0.16 g lunar gravity), 22.3° head up (0.38 g Mars gravity), and upright (1 g Earth gravity). Heart rate and respiration tidal volume increased as a function of the gravitational load, whereas respiration rate, and the volume of O_2_ and CO_2_ uptake decreased as a function of the gravitational loads.

## Body Loading/Unloading

In rodents, a 30° head-down tilt by tail suspension unloads the weight-bearing hindlimbs and creates a cephalad fluid shift and situational stress that are analogs to weightlessness (Morey-Holton and Globus, 2002). Ulanova et al. showed that a 7-days hindlimb suspension resulted in soleus muscle atrophy and a decrease in titin (T1), a protein responsible for the passive elasticity of muscle. Administration of L-Arginine, a semi-essential amino acid, decreased the degree of atrophy and prevented the decrease in T1 levels, thus mitigating the effects of gravitational unloading.

Mortreux et al. used a partial weight suspension system that allowed for reduced loading on all four limbs while still permitting quadrupedal locomotion (Wagner et al., 2010). Here, rats were supported for 14 days under Mars-analog suspension (38% weight bearing) and compared with age-matched (100% weight bearing) controls. Their results demonstrated that resveratrol, a polyphenol that is known to preserve bone and muscle mass, treatment during partial unloading significantly preserves muscle function and mitigates muscle atrophy.

Arntz et al. studied the vestibular drive for standing balance in humans when loading the body downward at 1.5 and 2 times their body weight, and when exposing subjects to hypergravity (1.8 g) during parabolic flights. A stochastic electrical vestibular stimulus (0–25 Hz) delivered during these tasks evoked a vestibular-error signal and corrective muscles responses. With additional load, the magnitude of the vestibular-evoked muscle responses progressively decreased and plateaued at 1.5 times body weight.

## Centrifugation

Diaz-Artiles et al. exposed subjects to 1.4 g at their feet via short-radius centrifugation and measured their cardiovascular responses while exercising on a cycle ergometer. They found that cardiac output, stroke volume, pulse pressure, and heart rate significantly increased with the gravity level. These results confirm that combination of exercise and artificial gravity provide a greater cardiovascular benefit than exercise alone.

## Author Contributions

All authors listed have made a substantial, direct and intellectual contribution to the work, and approved it for publication.

### Conflict of Interest

The authors declare that the research was conducted in the absence of any commercial or financial relationships that could be construed as a potential conflict of interest.
